# β-TCP from 3D-printed composite scaffolds acts as an effective phosphate source during osteogenic differentiation of human mesenchymal stromal cells

**DOI:** 10.3389/fcell.2023.1258161

**Published:** 2023-10-26

**Authors:** Luan P. Hatt, Daphne van der Heide, Angela R. Armiento, Martin J. Stoddart

**Affiliations:** ^1^ AO Research Institute Davos, Davos, Switzerland; ^2^ Institute for Biomechanics, ETH Zürich, Zürich, Switzerland

**Keywords:** β-tricalcium phosphate, 3D-printing, biomaterial, osteogenesis, mesenchymal stem cells, β-glycerophosphate replacement

## Abstract

**Introduction:** Human bone marrow-derived mesenchymal stromal cells (hBM-MSCs) are often combined with calcium phosphate (CaP)—based 3D-printed scaffolds with the goal of creating a bone substitute that can repair segmental bone defects*. In vitro*, the induction of osteogenic differentiation traditionally requires, among other supplements, the addition of β-glycerophosphate (BGP), which acts as a phosphate source. The aim of this study is to investigate whether phosphate contained within the 3D-printed scaffolds can effectively be used as a phosphate source during hBM-MSC *in vitro* osteogenesis.

**Methods:** hBM-MSCs are cultured on 3D-printed discs composed of poly (lactic-co-glycolic acid) (PLGA) and β-tricalcium phosphate (β-TCP) for 28 days under osteogenic conditions, with and without the supplementation of BGP. The effects of BGP removal on various cellular parameters, including cell metabolic activity, alkaline phosphatase (ALP) presence and activity, proliferation, osteogenic gene expression, levels of free phosphate in the media and mineralisation, are assessed.

**Results:** The removal of exogenous BGP increases cell metabolic activity, ALP activity, proliferation, and gene expression of matrix-related (*COL1A1, IBSP, SPP1*), transcriptional (*SP7, RUNX2/SOX9, PPARγ*) and phosphate-related (*ALPL, ENPP1, ANKH, PHOSPHO1*) markers in a donor dependent manner. BGP removal leads to decreased free phosphate concentration in the media and maintained of mineral deposition staining.

**Discussion:** Our findings demonstrate the detrimental impact of exogenous BGP on hBM-MSCs cultured on a phosphate-based material and propose β-TCP embedded within 3D-printed scaffold as a sufficient phosphate source for hBM-MSCs during osteogenesis. The presented study provides novel insights into the interaction of hBM-MSCs with 3D-printed CaP based materials, an essential aspect for the advancement of bone tissue engineering strategies aimed at repairing segmental defects.

## 1 Introduction

Reconstructing large segmental mandibular bone defects using autologous bone grafting is met with several disadvantages such as limited availability or donor site morbidities (Paré et al., 2019). Tissue engineered bone substitutes have emerged as a promising alternative with the aim to replace autologous bone grafting as the standard of care (Hatt et al., 2022a). Human bone marrow-derived mesenchymal stromal cells (hBM-MSCs) are multipotent cells that possess the ability to differentiate into bone forming osteoblasts, making them a promising candidate for the treatment of bone-related conditions (Kemp et al., 2005). Consequently, osteogenically driven hMSCs are often used in combination with calcium-phosphate (CaP)-based 3D-printed scaffolds to create a bone substitute, offering a solution for bone defect repair (Khojasteh et al., 2013; Probst et al., 2020; Rong et al., 2020; Tu et al., 2020). The introduction of 3D printing of bone substitutes provided numerous advantages for bone tissue engineering such as creating patient-specific scaffolds, tailored design patterns for improved tissue guidance and tunable porosity to facilitate cell and tissue infiltration. Among the various biomaterials used for scaffold fabrication, composites have attracted significant attention, due to the possibility to combine diverse properties from different materials such as CaP and polymers (van der Heide et al., 2022). β-tricalcium phosphate (β-TCP) is a CaP-based ceramic, that provides osteoconductivity and biodegradability, resembling the mineral phase of natural bone tissue (Yamada et al., 2007) and has been shown to support the clinical integration into tissue engineered bone substitutes (Thesleff et al., 2011; Sándor et al., 2013; Sándor et al., 2014; Manimaran et al., 2016). However, it lacks optimal mechanical stability (Liu and Lun, 2012; Salamanca et al., 2021), which can be overcome by incorporating a polymer such as poly (lactic-co-glycolic acid) (PLGA) (Kang et al., 2011; Zhao et al., 2021).

Before clinical translation of a bone substitute can occur, it is crucial to assess and validate its osteogenic capacity. *In vitro* osteogenesis of MSCs serves as initial step in this validation process (Bongio et al., 2015). The traditional *in vitro* induction of osteogenesis requires medium supplementation with a differentiation cocktail that includes dexamethasone, ascorbic acid and organic β-glycerophosphate (BGP) (Hatt et al., 2022b). This osteogenic cocktail induces cell morphology changes, increases alkaline phosphatase (ALP) activity, the expression of osteogenic gene markers and secretion of matrix minerals (Langenbach and Handschel, 2013; Zha et al., 2022). BGP acts as a phosphate source to be cleaved by MSCs resulting in the release of phosphate ions needed for the cells to produce and secrete hydroxyapatite (Tenenbaum, 1981; Maniatopoulos et al., 1988; Nantavisai et al., 2019; Zha et al., 2022). However, high phosphate levels are known to have toxic effects in several cell types including embryonic kidney (HEK) 293 (He et al., 2021), epithelial HeLa (He et al., 2021) and EA hy926 endothelial cells (Di Marco et al., 2013). Hyperphosphatemia causes vascular calcification of vascular smooth muscle cells, which is associated with cardiovascular diseases (Giachelli, 2009), as well as metabolic bone diseases (Zhou et al., 2021; Anand and Aoyagi, 2023). MSCs are sensitive to exogenous phosphate ions and optimal phosphate levels are known to be important for osteoblast differentiation (Beck, 2003; Liu et al., 2009). BM-MSC proliferation and differentiation is decreased with greater or lower concentration of inorganic phosphate supplementation, with greater concentrations leading to cell apoptosis (Liu et al., 2009). The cellular and molecular mechanism, by which elevated phosphate alters cell behavior, remains unclear (Camalier et al., 2013). Therefore, caution must be taken when using exogenous BGP in medium supplementation.

Replacing BGP with inorganic phosphate as an alternative phosphate source has been shown to stabilize free phosphate levels in the culture medium, to improve the quality of hydroxyapatite secreted by MSCs (Schäck et al., 2013) and to enhance the mineral deposition outcome (Hatt et al., 2019). Osteogenic differentiation of MSCs cultured on a 3D-printed phosphate-based scaffold using BGP as the phosphate source has been investigated in several studies: adipose-derived MSCs cultured on 3D-printed PLGA/TCP (Probst et al., 2020) scaffold, polycaprolactone/hydroxyapatite (PCL/HAp) scaffold (Nyberg et al., 2017; Kuss et al., 2018; Ebrahimi et al., 2022) or PCL/TCP (Nyberg et al., 2017) scaffold, as well as BM-MSCs cultured on 3D-printed PLGA/CaP cement scaffold (Qian et al., 2019), β-TCP scaffold (Jiao et al., 2022; Meesuk et al., 2022; Hatt et al., 2023) or polylactic acid/HAp scaffold (Bernardo et al., 2022). However, the ability of MSCs to use inorganic phosphate contained within the 3D-printed scaffolds during *in vitro* osteogenesis is poorly understood. Hence, this study aims to investigate the potential of β-TCP embedded within 3D-printed PLGA/β-TCP scaffolds to serve as an effective phosphate source during the osteogenic differentiation of hBM-MSCs. Through systematic osteogenic *in vitro* experiments, we evaluate the proliferation and osteogenic differentiation by examining key osteogenic markers of hBM-MSCs cultured on 3D-printed PLGA/β-TCP discs. Understanding the underlying interactions between bone substitutes and MSC behavior is essential for the advancement of bone tissue engineering strategies aimed at repairing bone defects.

## 2 Materials and methods

**FIGURE 1:**
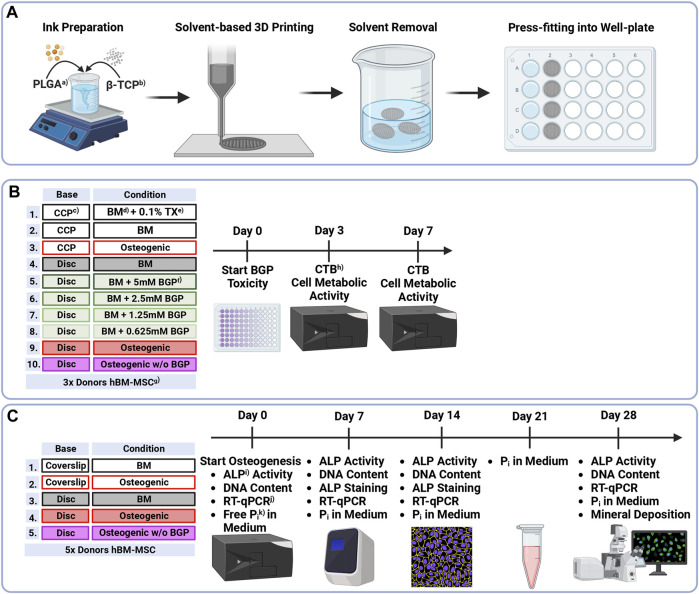
Methodical Overview of **(A)** Preparation, solvent-based 3D printing and postprocessing of PLGA/β-TCP discs, **(B)** Groups and timeline of β-glycerophosphate cytotoxicity assessment of hBM-MSCs cultured on either culture-plate plastic or 3D-printed PLGA/β-TCP discs for 7 days under different condition measured using the CellTiter-Blue^®^ Cell Viability Assay and **(C)** Groups and timeline of osteogenic differentiation assessment of hBM-MSC cultured on either coverslips or 3D-printed discs for 28 days under different conditions measuring ALP Activity, DNA content, ALP staining, gene expression via RT-qPCR, free phosphate concentration in the medium and mineral deposition via OsteoImage^®^ staining. ^a)^PLGA = as poly (lactic-co-glycolic acid), ^b)^β-TCP = β-tricalcium phosphate, ^c)^CCP = cell-culture plastic, ^d)^BM = basal medium, ^e)^TX = Triton X-100, ^f)^BGP = β-glycerophosphate, ^g)^hBM-MSCs = human bone marrow-derived mesenchymal stromal cells, ^h)^CTB = CellTiter-Blue^®^, ^i)^ALP = alkaline phosphatase, ^j)^RT-qPCR = reverse transcription-quantitative polymerase chain reaction, ^k)^P_i_ = phosphate.

Human bone marrow aspirates are obtained with informed consent of all donors and with full approval from the Ethics Committee of the University of Freiburg Medical Centre (EK-Freiburg: 135/14, 25 March 2014) and the ethical commission of Zürich (KEK-ZH-NR: 2016-00141). All reagents are purchased from Sigma-Aldrich unless otherwise stated. An overview of the methods is reported in Figure 1.

### 2.1 Preparation, solvent-based printing and postprocessing of PLGA/β-TCP discs

Solvent-based printing and postprocessing are performed as previously described (Hatt et al., 2023). (PURASORB^®^ PLG 8531, 85/15 L-lactic/Glycolide, Corbion) powder is dissolved in ethylene carbonate at 90°C using a hot plate stirrer and a Hei-TORQUE Expert 200 (Heidolph Instruments) mixing system set to 30 rpm. Subsequently, the system is cooled to 80°C, β-TCP powder (BABI-TCP-N100, Berkeley Advanced Biomaterials) is added, and the blend stirred overnight. The ink is transferred to 3 cc syringe barrels (Nordson EFD) and kept at −20°C until printing. Low-temperature solvent-based 3D printing is applied to the ink-laden cartridges equipped with a 0.5 mm stainless steel needle using a 3D Discovery^®^ bioprinter (RegenHU). The ink is extruded onto a dry glass slide which is mounted onto an aluminium cool plate according to printing parameters shown in Table 1. Dense 3D-printed discs with a total height of 0.8 mm (2 layers) and a diameter of 19 mm are printed. Subsequently, discs are placed in a vacuum oven at room temperature for 24 h and then in a water bath at 37°C for 48 h (4x Milli-Q^®^ water changes) to extract the solvent. Total solvent extraction is confirmed via 1H nuclear magnetic resonance. Upon air drying at room temperature, discs are sterilised using a cold ethylene oxide gas protocol, degassed under vacuum, and cut using a 13 mm or 7.5 mm diameter puncher to be press-fit into a 24 cell-culture plate or a 96 well-plate, respectively. All steps are presented in Figure 1A.

**TABLE 1:** Solvent-based printing parameters.

Cartridge heater (°C)	Pressure	Velocity	Layer height (mm)	Cool plate
80	3.5–4 bar	4 mm/s	0.4	3°C–10°C

### 2.2 Cell isolation and culture of hBM-MSCs

Isolation and cryopreservation of hBM-MSCs from bone marrow aspirates are conducted following published protocols (Armiento et al., 2023). Cell culture expansion was performed following the protocols as previously described (Hatt et al., 2019). In short, upon thawing, 0.9 × 10^6^ cells are seeded in a T300 flask (cell density 3 × 10^3^ cells/cm^2^) for culture expansion. The expansion medium consists of α-MEM (Gibco) supplemented with 10% (v/v) foetal bovine serum (FBS) (Corning) and 100 U/mL Penicillin, 100 μg/mL Streptomycin (PEN/STREP) (Gibco). Cells are cultured under standard cell culture conditions of 37°C with 5% CO_2_ and 90% humidity with 3 media changes per week before they are used for the BGP cytotoxicity assessment (Figure 1B) or osteogenic differentiation experiment (Figure 1C). hBM-MSCs donor details are as follows: Donor A, 24-year old male, iliac crest, Donor B, 73-year old female, spine vertebral body; Donor C, 54-year old male, hip cancellous bone; Donor D, 48-year old female, spine vertebral body, Donor E, 61-year old female, hip cancellous bone; Donor F, 51-year old female, spine vertebral body; Donor G, 74-year old female spine vertebral body; Donor H, 71-year old female, spine vertebral body.

### 2.3 Cytotoxicity of BGP on hBM-MSCs via CellTiter-Blue^®^ assessment

Cytotoxicity of BGP on hBM-MSCs when seeded on either cell-culture plastic (CCP) or 3D-printed PLGA/β-TCP discs that are press fitted into well plates is carried out using the CellTiter-Blue^®^ (CTB) Cell Viability Assay (Promega) performed according to the company’s instructions (Figure 1B). Cells are harvested using 0.05% Trypsin-EDTA (Gibco) and seeded in 96-well plates at a density of 1 × 10^4^ cells/well in 4 replicates. For the first 24 h, cells are cultured in basal medium (BM) (LG-DMEM (Gibco) supplemented with 10% (*v/v*) FBS and 1% (*v/v*) PEN/STREP. At this point (Day 0), cells are switched into their corresponding culture media: cells cultured on CCP 1) Positive control: BM supplemented with 0.1% Triton X-100; 2) Negative control: BM; 3) Osteogenic: BM supplemented with 10 nM dexamethasone, 5 mM BGP and 50 μg/mL L-ascorbic acid-2-phosphate; cells cultured on discs: 4) BM; 5) BM supplemented with 5 mM BGP; 6) 2.5 mM BGP; 7) 1.25 mM BGP; 8) 0.625 mM BGP; 9) Osteogenic and 10) Osteogenic w/o BGP: BM supplemented with 10 nM dexamethasone and 50 μg/mL L-ascorbic acid-2-phosphate. Cell metabolic activity measurement is performed at day 3 and day 7. Media are removed and BM supplemented with 16.6% (v/v) CTB reagent is added. After 4 h of incubation at 37°C with 5% CO_2_ and 90% humidity the supernatant is transferred into a 96 clear bottom well-plate (Corning) and fluorescence is read at 560/590 nm using Infinite^®^ 200 PRO plate reader (Tecan Trading AG).

### 2.4 Osteogenic differentiation of hBM-MSCs

hBM-MSCs are harvested using 0.05% Trypsin-EDTA and seeded at a density of 28.5 × 10^3^ cells/well (15 × 10^3^ cells/cm^2^) in duplicates onto either coverslips (SARSTEDT AG) or PLGA/β-TCP 3D-printed discs that are press fitted into a 24-well plate (Figure 1C). Media compositions are presented in Table 2. For the first 24 h, cells are cultured in BM and subsequently switched into their corresponding media. The experiment is performed under 5 different conditions: 1) coverslip BM; 2) coverslip osteogenic; 3) disc BM; 4) disc osteogenic and 5) disc osteogenic without BGP for 28 days under standard culture conditions.

**TABLE 2:** Media composition.

	Basal medium	Osteogenic	Osteogenic w/o BGP
LG-DMEM	+	+	+
PEN/STREP	+	+	+
FBS	+	+	+
Dexa	-	+	+
AA2P	-	+	+
BGP	-	+	-

LG-DMEM: low glucose (1 g/L) DMEM, PEN/STREP: Penicillin/Streptomycin, FBS: fetal bovine serum, Dexa: dexamethasone, AA2P: L-ascorbic acid 2-phosphate sesquimagnesium salt hydrate, BGP: β-Glycerophosphate disodium salt hydrate.

### 2.5 Quantification of ALP activity and DNA content

At day 0, 7, 14 and 28, ALP activity and DNA content are measured as previously described (Hatt et al., 2019) (Figure 1C). In short, after cell lysis with 0.1% Triton X-100 in 10 mM TrisHCl, the enzymatic reaction is started by adding alkaline buffer solution, substrate solution (25 mg/mL phosphate substrate in 1 mM diethanolamine) and Milli-Q^®^ water and stopped by adding 0.1 M NaOH solution after 15 min at 37°C. The absorbance is read at 405 nm using the Infinite^®^ 200 PRO plate reader. ALP activity is normalised to the DNA content. DNA concentration is quantified at day 0, 7, 14 and 28 using the CyQuant™ Cell Proliferation Assay (Invitrogen) according to the manufacturer’s instructions. Cell lysate is transferred into a 96 clear bottom well plate, working solution containing dye is added, incubated for 5 min and fluorescence is read at 490/530 nm using Infinite^®^ 200 PRO plate reader.

### 2.6 ALP staining

At day 7 and 14 ALP is stained using the alkaline phosphatase staining kit (Procedure No. 85) according to the company’s instructions (Figure 1C). In short, cells are washed 3x with phosphate buffered saline (PBS), fixed with 10% neutral buffered formalin for 30 min and, after 3x Milli-Q^®^ water rinses, stained with the alkaline dye solution for 30 min at room temperature. 50 mL alkaline dye solution is composed of one Fast Blue RR Salt capsule and 2 mL Naphthol AS-MX Phosphate Alkaline solution. Upon water rinsing the samples are imaged.

### 2.7 RNA isolation and reverse transcription-quantitative polymerase chain reaction (RT-qPCR)

Cells are harvested for gene expression analysis at day 0, 7, 14 and 28. RNA isolation and RT-qPCR is performed using the QuantStudio™ Flex Real-Time PCR System as previously described (Hatt et al., 2019) (Figure 1C). Reverse transcription is performed using the Superscript Vilo cDNA Synthesis Kit (Thermo Fisher Scientific) according to the company’s instructions. *ALPL* (encodes for ALP), *ANKH* (encodes for progressive ankylosis protein homolog), *COL1A1* (encodes for alpha-1 type 1 collagen), *ENPP1* (encodes for ectonucleotide pyrophosphatase/phosphodiesterase family member 1), *IBSP* (encodes for bone sialoprotein), *PHOSPHO1* (encodes for phosphoethanolamine/phosphocholine phosphatase 1), *PPARγ* (encodes for peroxisome proliferator-activated receptor gamma), *RUNX2* (encodes for runt-related transcription factor 2), *SP7* (encodes osterix), *SPP1* (encodes osteopontin) and *SOX9* (encodes for SRY-box transcription factor 9) gene expressions are investigated. Primer sequences used are listed in Table 3. The 2^−ΔΔCT^ method is applied for data analysis using *RPLP0* as an endogenous normaliser and day 0 samples as a calibrator.

**TABLE 3:** Primers/probes used for RT-qPCR.

Gene	Assay on demand^a^
*ALPL*	*Hs00758162_m1*
*ANKH*	*Hs01064613_m1*
*ENPP1*	*Hs01054040_m1*
*IBSP*	*Hs00173720_m1*
*PHOSPHO1*	*Hs01370290_m1*
*PPARγ*	*Hs00234592_m1*
*SP7*	*Hs00541729_m1*
*SPP1*	*Hs00959010_m1*
*SOX9*	*Hs00165814_m1*

**Gene**	**Forward**	**Reverse**	**Probe**
*COL1A1*	*5′-CCC TGG AAA GAA TGG AGA TGA T-3′*	*5′-ACT GAA ACC TCT* *GTG TCC CTT CA-3′*	*5′-CGG GCA ATC CTC* *GAG CAC CCT-3′*
*RPLP0*	*5′-TGG GCA AGA ACA CCA TGA TG-3′*	*5′-CGG ATA TGA GGC AGC AGT TTC-3′*	*5′-AGG GCA CCT GGA AAA CAA CCC AGC-3′*
*RUNX2*	*5′-AGC AAG GTT CAA CGA TCT GAG AT-3′*	*5′-TTT GTG AAG ACG* *GTT ATG GTC AA-3′*	*5′-TGA AAC TCT TGC* *CTC GTC CAC TCC G-3′*

*ALPL*: alkaline phosphatase, biomineralisation associated; *IBSP*: integrin binding sialoprotein; *SP7*: Sp7 transcription factor; *RPLP0*: ribosomal protein lateral stalk subunit P0; *SPP1:* osteopontin, organic component of bone matrix.

^a^ TaqMan^®^ Gene Expression Assay (Applied Biosystems^®^).

### 2.8 Free phosphate quantification

Levels of free phosphate are assessed in conditioned media once per week (Figure 1C) using the Quanticrome Phosphate Assay Kit (DPI-500, BioAssay Systems) according to the company’s instructions. Conditioned media are collected at day 0, 7, 14, 21 and 28 and stored at—20 °C prior to analysis.

### 2.9 Staining of mineral deposition

At day 28, cells are washed 3x with PBS, fixed with 10% neutral buffered formalin for 30 min, rinsed 3x with Milli-Q^®^ water, permeabilised with 0.25% Triton X-100 in Milli-Q^®^ water for 20 min and stained with 2 μg/mL 4′,6-Diamidino-2-phenylindole solution for 10 min with a Milli-Q^®^ water wash in between each step (Figure 1C). Mineral deposition is stained with OsteoImage™ Mineralization Assay (Lonza) according to the manufacturer’s instructions and imaging is performed using a confocal microscope (LSM800, Leica Microsystems).

### 2.10 Statistics

Statistical analyse is performed using GraphPad Prism software version 9.3.1 (GraphPad Software). A One-Way ANOVA is applied to the data of the cell metabolic activity. A Two-Way ANOVA is applied to the data of the ALP activity, DNA quantification, gene expression and free phosphate quantification. *p*-values lower than 0.05 are considered significant and thus marked.

## 3 Results

### 3.1 Exogenous BGP decreases cell metabolic activity of hBM-MSCs cultured on 3D-printed PLGA/β-TCP discs in a dose-responsive manner

**FIGURE 2:**
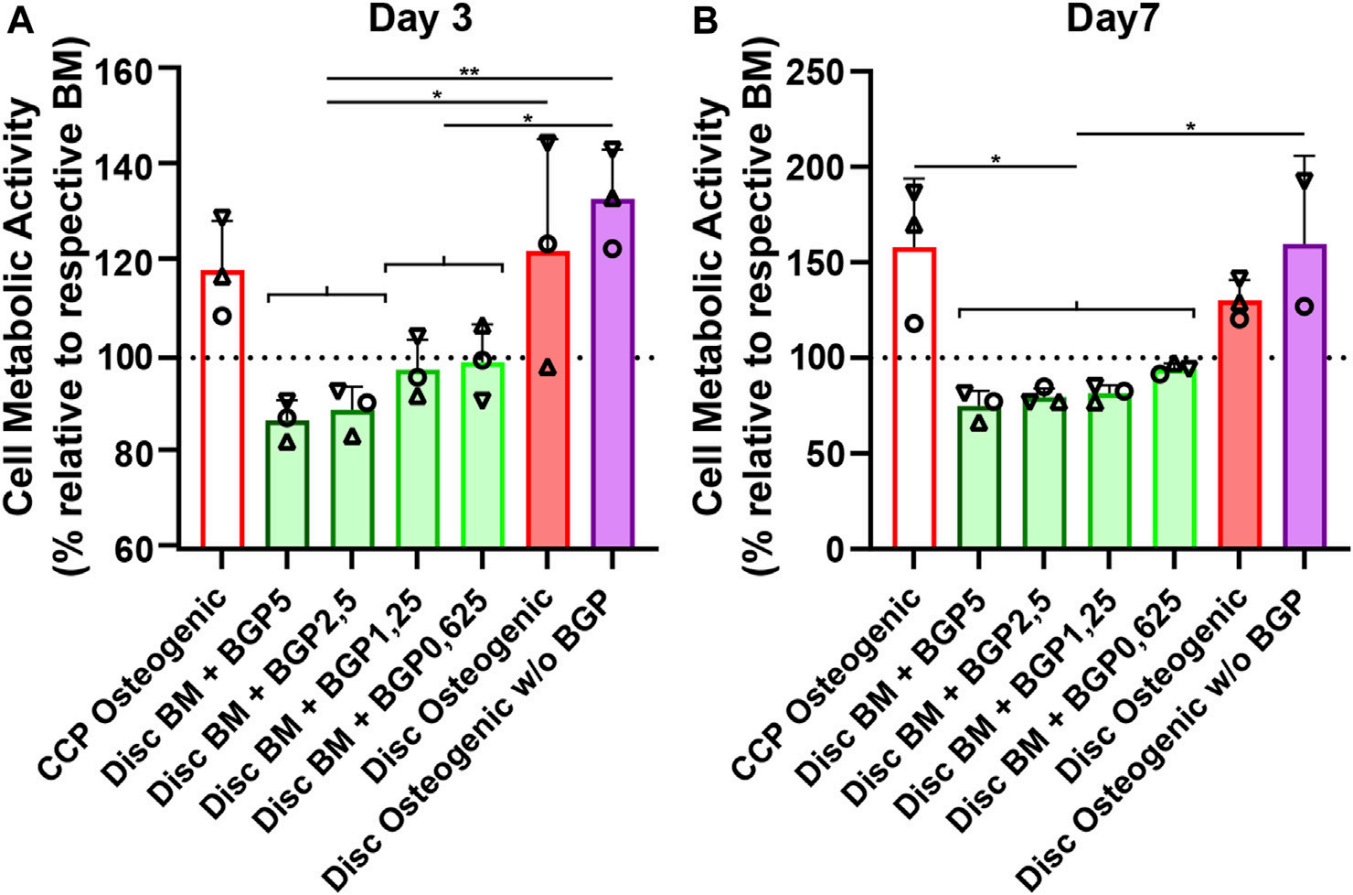
Cytotoxicity of β-glycerophosphate (BGP) on hBM-MSC cultured either on cell-culture plastic (CCP) or 3D-printed PLGA/β-TCP discs of three independent donors (N = 3). Individual data points shown are the mean of three technical replicates for each individual donor: donor A (○), donor B (△), donor C (▽). Cells are cultured in basal medium (BM), BM supplemented with 5 mM, 2,5 mM, 1,25 mM or 0,625 mM of BGP, osteogenic medium or osteogenic medium without BGP for 7 days. Cell metabolic activity is measured at **(A)** day 3 and **(B)** day 7 using the CellTiter-Blue^®^ Cell Activity Assay with the corresponding BM as normaliser. One-way ANOVA is performed: *p < 0.5, **p < 0.01.

hBM-MSCs of three independent donors cultured on either culture-plate plastic or 3D-printed PLGA/β-TCP discs for 7 days under either BM, BM with different concentrations of BGP or osteogenic medium with and without the standard concentration of 5 mM BGP (Figure 2). Cell metabolic activity measurements reveal a decrease of approximately 13% and 25% on day 3 and 7, respectively, in BM supplemented with 5 mM BGP compared to the corresponding BM set to 100%. A trend of dose-responsive increase of cell activity is measured when BGP concentrations are decreased from 5 mM to 0.625 mM. Cell metabolic activity of cells cultured under osteogenic condition without BGP supplementation is significantly increased compared to all BM groups supplemented with BGP at day 3 and 7 and insignificantly increased compared to the osteogenic group.

BM supplementation with exogenous BGP decreases cell activity of hBM-MSCs when cultured on 3D-printed PLGA/β-TCP discs in a dose-responsive manner and results in cytotoxic-like consequences.

### 3.2 Absence of exogenous BGP increases presence and activity of ALP and proliferation of hBM-MSCs cultured on 3D-printed PLGA/β-TCP discs under osteogenic conditions

**FIGURE 3:**
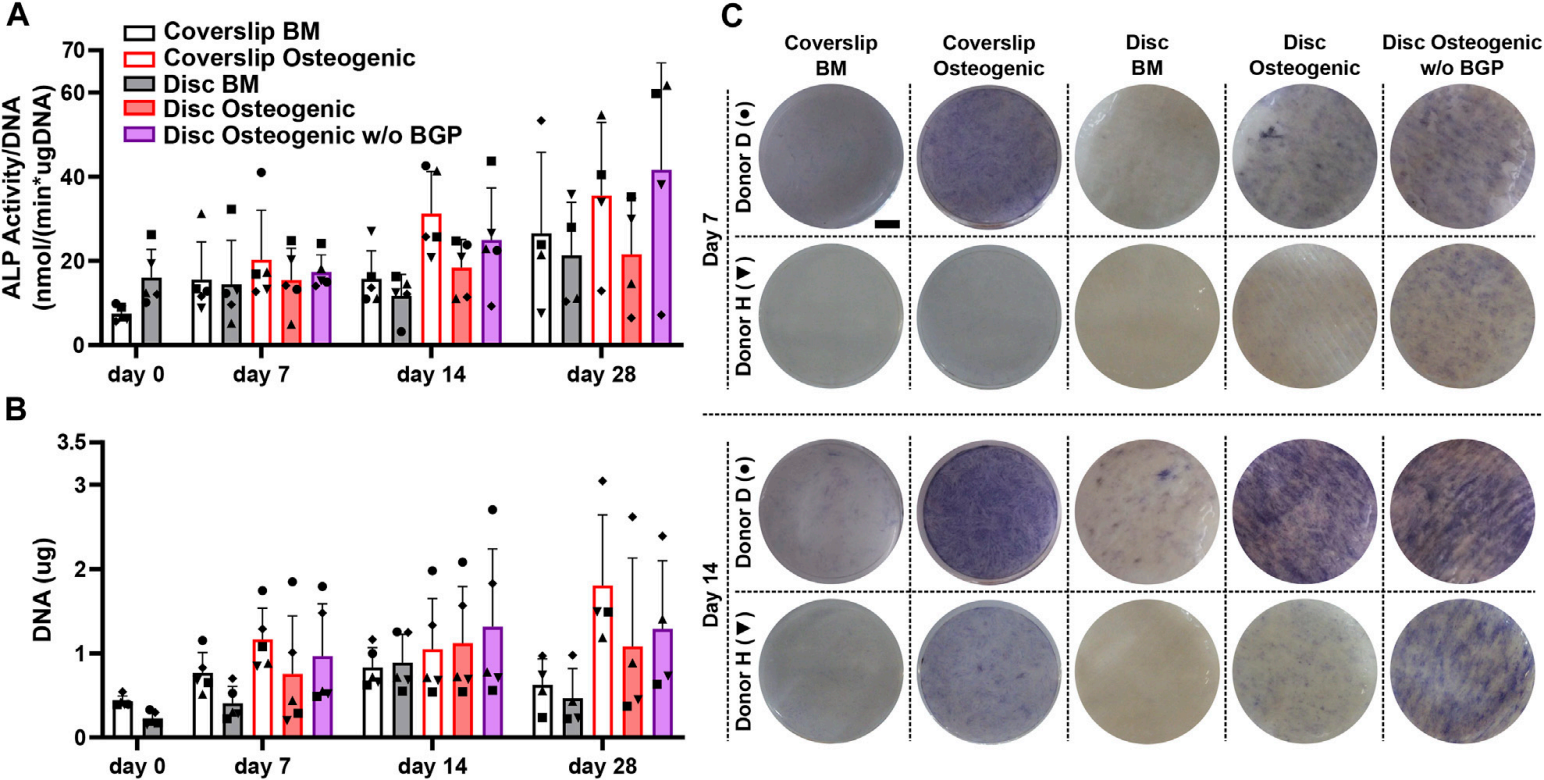
Alkaline phosphatase (ALP) and proliferation assessment of hBM-MSC cultured either on coverslips or 3D-printed PLGA/β-TCP discs of five independent donors (N = 5). Individual data points shown are the mean of two technical replicates for each individual donor: donor D (●), donor E (▲), donor F (♦), donor G (■) and donor H (▼). Cells are cultured in basal medium (BM), osteogenic medium or osteogenic medium without β-glycerophosphate (BGP) for 28 days. **(A)** ALP activity normalised to DNA content at day 0, 7, 14 and 28; **(B)** Images of ALP staining of two representative donors: donor D (●) and donor H (▼) at day 7 and 14, scale bar 2, 5 mm; **(C)** DNA content at day 0, 7, 14 and 28.

The ALP activity measurement normalised to DNA content, shows a trend of mean upregulation of osteogenically driven cells cultured on the 3D-printed discs at day 14 and no difference at day 7 and 28 (Figure 3A). The removal of BGP from the osteogenic medium slightly increases the trend of mean ALP activity at day 7 and more profoundly at day 14 and 28 compared to the osteogenic group. ALP activity seems to be delayed of osteogenically driven cells cultured on the disc to day 28, while cells cultured on coverslips peak at day 14. ALP staining confirms the protein expression at day 7 and 14 shown by two representative donors (donor D and H). Cells cultured on 3D-printed discs visibly show increased stained area of ALP at day 7 and 14 in the osteogenic group compared to the BM group (Figure 3B). The removal of BGP even further enhances ALP staining at day 7 and 14 in donor H and maintains the stained area for donor D compared to the osteogenic group. The degree of ALP staining is in accordance with the protein expression for each donor from the ALP activity profile. Osteogenic treatment increases the trend of mean proliferation of the cells at all time points when cultured on coverslips or 3D-printed discs (Figure 3C). The subtraction of BGP further increases the proliferation compared to the osteogenic group.

The absence of exogenous BGP leads to an overall increase in ALP activity and staining, as well as enhanced proliferation of hBM-MSCs cultured on 3D-printed PLGA/β-TCP discs.

### 3.3 Absence of exogenous BGP upregulates or maintains gene expression of osteogenic markers involved in matrix production of hBM-MSCs cultured on 3D-printed PLGA/β-TCP discs under osteogenic conditions

**FIGURE 4:**
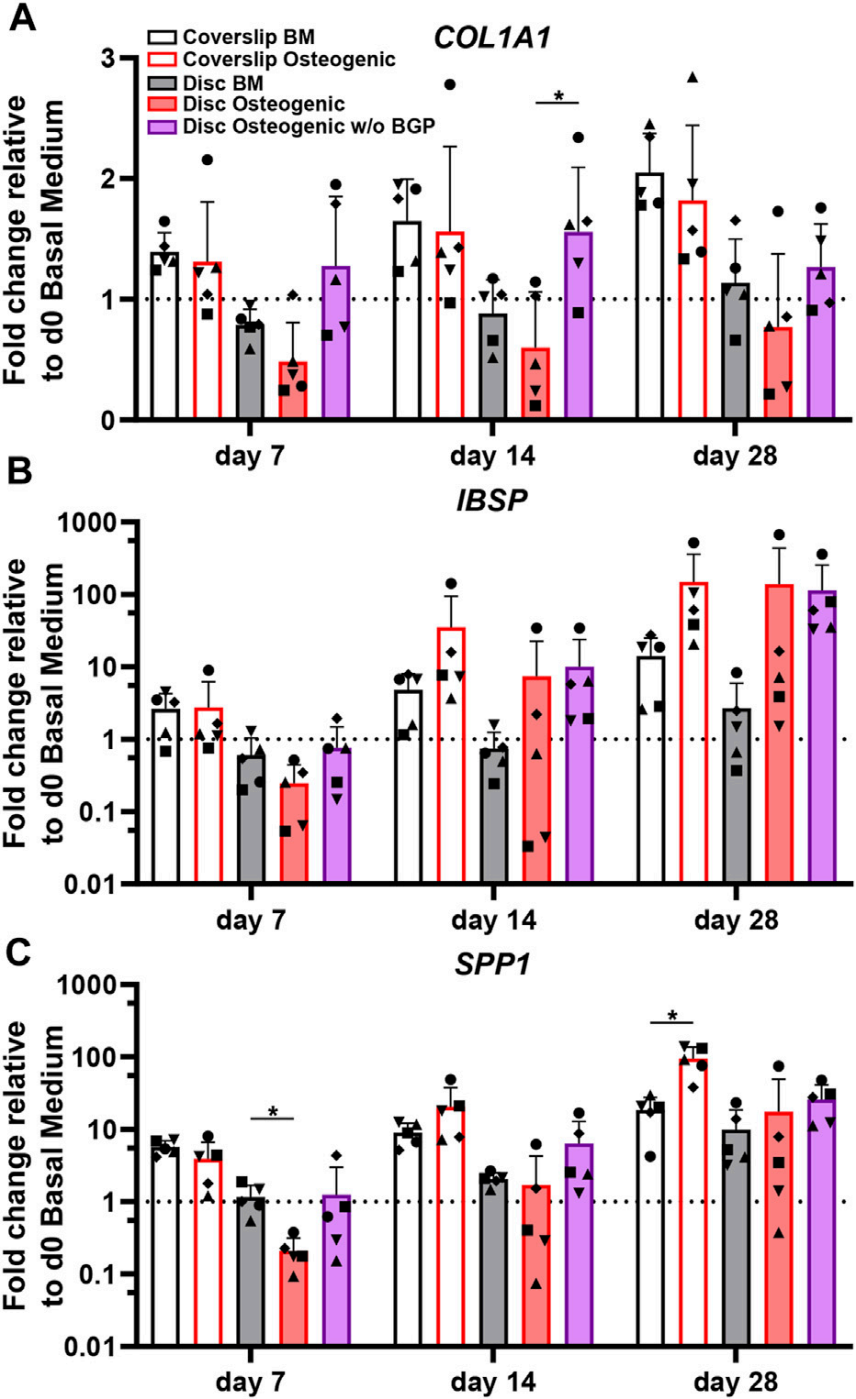
Gene expression of osteogenic markers involved in matrix production of hBM-MSC cultured either on coverslips or 3D-printed PLGA/β-TCP discs of five independent donors (N = 5). Individual data points shown are the mean of two technical replicates for each individual donor: donor D (●), donor E (▲), donor F (♦), donor G (■) and donor H (▼). Cells are cultured in basal medium (BM), osteogenic medium or osteogenic medium without β-glycerophosphate (BGP) for 28 days. Fold changes in **(A)** *Col1A1,* **(B)** *IBSP* and **(C)** *SPP1* expression at day 7, 14 and 28 are calculated according to the 2^−ΔΔCt^ method using *RPLP0* as the endogenous calibrator and the corresponding day 0 BM the normaliser. Two-way ANOVA is performed: *p < 0.05.

*COL1A1*, which encodes for alpha-1 type 1 collagen, an organic protein necessary for the formation bone tissue, is an important early osteogenic marker for matrix production of osteogenically driven hBM-MSCs. The fold change gene expression, relative to the corresponding day 0 BM of five independent donors cultured on 3D-printed discs under standard osteogenic conditions, is downregulated compared to the BM groups for all time points (Figure 4A). The subtraction of BGP causes the expression of *COL1A1* to rise compared to the osteogenic medium with statistical significance at day 14 from 0.6 ± 0.46 to 1.56 ± 0.53 (*p* = 0.39) and without statistical significance at day 7 and 28. Applying the standard osteogenic cocktail to the cells slightly decreases the trend of mean fold change of *IBSP* gene expression, the bone sialoprotein encoding gene, a late stage osteoblast differentiation marker for matrix production at day 7, but increases at day 14 and 28 (Figure 4B). The removal of BGP upregulates the mean fold change gene expression of *IBSP* at day 7 and maintains it at day 14 and 18 and compared to the osteogenic group. The fold change of *SPP1*, the osteopontin encoding gene, a late osteogenic marker involved in matrix regulation, is significantly downregulated in the osteogenic group at day 7 compared to the BM from 1.16 ± 0.53 to 0.21 ± 0.11 (*p* = 0.0311), which recovers over time and the trend increases at day 28 (Figure 4C). The subtraction of BGP increases the *SPP1* fold change expression trend at day 7, 14 and 28 compared to the osteogenic medium.

The absence of exogenous BGP leads to an overall trend of upregulated gene expression levels relevant to matrix production of hBM-MSCs cultured on 3D-printed PLGA/β-TCP discs.

### 3.4 Absence of exogenous BGP upregulates or maintains gene expression of transcription factors involved in the differentiation of hBM-MSCs cultured on 3D-printed PLGA/β-TCP discs under osteogenic conditions

**FIGURE 5:**
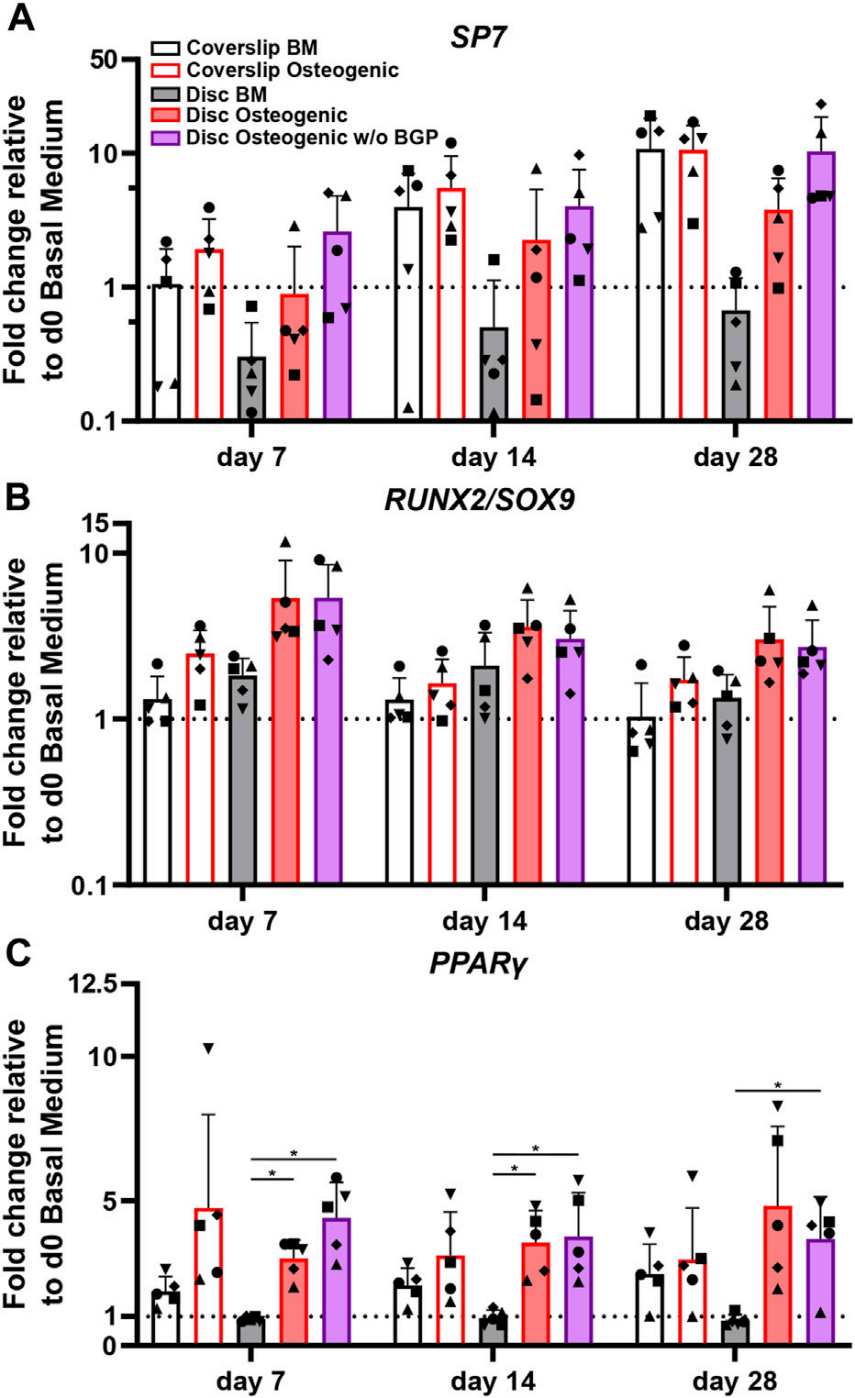
Gene expression of transcription factors involved in MSC differentiation fate of hBM-MSC cultured either on coverslips or 3D-printed PLGA/β-TCP discs of five independent donors (N = 5). Individual data points shown are the mean of two technical replicates for each individual donor: donor D (●), donor E (▲), donor F (♦), donor G (■) and donor H (▼). Cells are cultured in basal medium (BM), osteogenic medium or osteogenic medium without β-glycerophosphate (BGP) for 28 days. Fold changes in **(A)** *SP7,* **(B)** *RUNX2/SOX9* ratio and **(C)** *PPARγ* expression at day 7, 14 and 28 are calculated according to the 2^−ΔΔCt^ method using *RPLP0* as the endogenous calibrator and the corresponding day 0 BM the normaliser. Two-way ANOVA is performed: *p < 0.5, * *p < 0.01.

*SP7,* the osterix encoding gene, is an important transcription factor and driver of MSCs differentiation into osteoblasts and therefore used as an osteogenic marker for hBM-MSC osteogenesis. The *SP7* fold change gene expression trend of cells cultured on 3D-printed discs show a positive response under osteogenic treatment compared to BM (Figure 5A). The removal of BGP further enhances the *SP7* fold change expression trend at day 7, 14 and 28 compared to the osteogenic medium. The ratio between *RUNX2* and *SOX9* transcription factors is relevant for MSC osteogenesis and chondrogenesis, respectively and therefore also used as an osteogenic predictor for the differentiation of hBM-MSCs. Osteogenically driven cells show an increased trend of fold change ratio compared to BM, which is maintained when the BGP is subtracted from the osteogenic medium (Figure 5B). *PPARγ* is a transcription factor that drives MSC adipogenesis, the third possible linage of MSCs differentiation (Della Bella et al., 2021). Cells cultured under osteogenic conditions with or without the supplementation of BGP show a significant upregulation of the fold change expression of *PPARγ* compared to BM from 0.91 ± 0.089 to 3 ± 0.65 or 4.41 ± 1.23, respectably (*p* = 0.004, *p* = 0.0068, respectively) at day 7 and from 0.96 ± 0.27 to 3.96 ± 1.1 or 3.77 ± 1.52, respectively (*p* = 0.0114, *p* = 0.0292, respectively) at day 14, while the change at day 28 is only significant for the osteogenic group without BGP from 0.86 ± 0.2 to 3.68 ± 1.46 (*p* = 0.0262) (Figure 5C). The removal of BGP leads to increased trend of expression levels of *PPARγ* compared to the osteogenic group at day 7, maintained at day 14 and decreased at day 28.

The absence of exogenous BGP leads to an overall maintenance or upregulation of genes involved in the differentiation pathways of hBM-MSCs cultured on 3D-printed PLGA/β-TCP discs.

### 3.5 Absence of exogenous BGP upregulates and recovers gene expression of phosphate relevant markers of hBM-MSCs cultured on 3D-printed PLGA/β-TCP discs under osteogenic conditions

**FIGURE 6:**
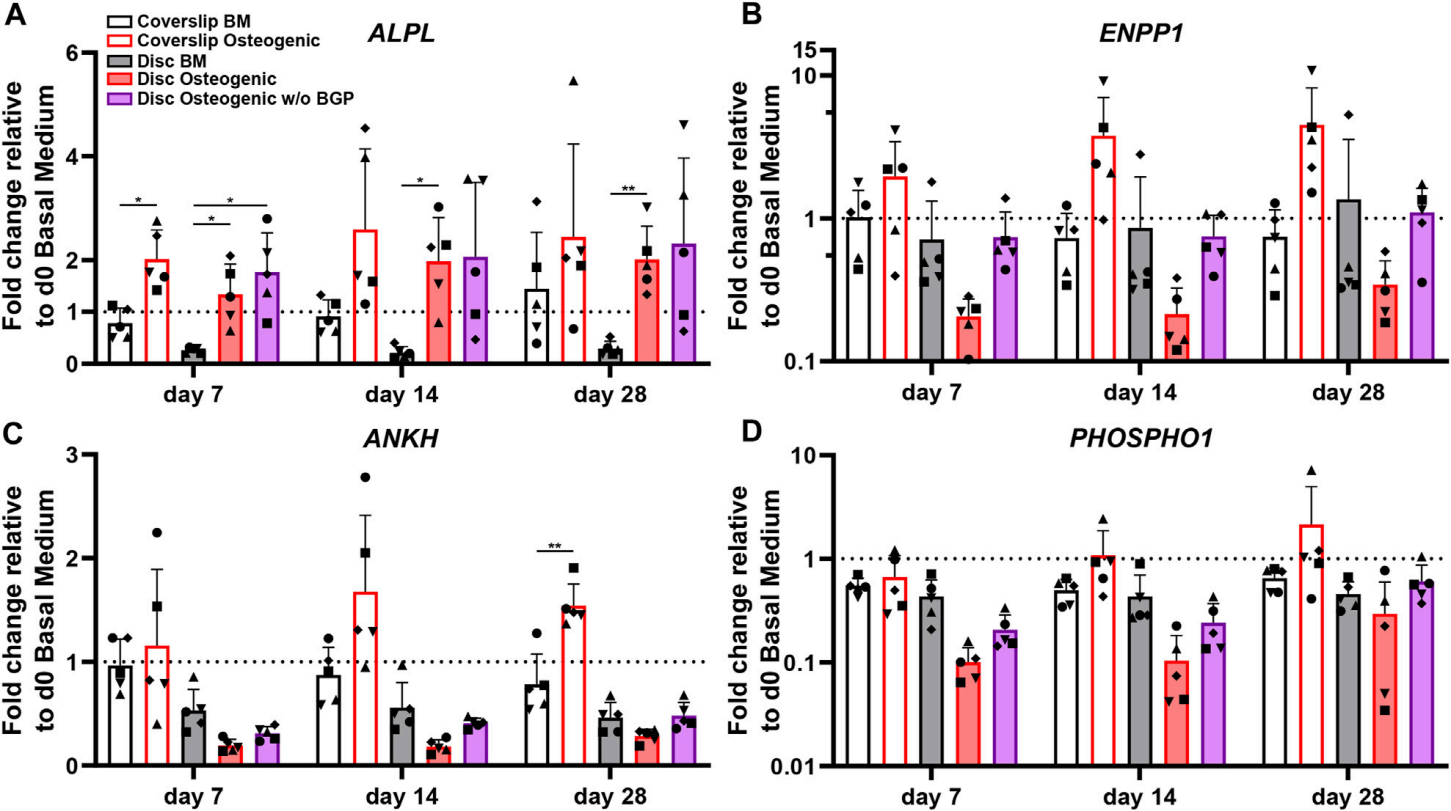
Gene expression of phosphate-related markers of hBM-MSC cultured either on coverslips or 3D-printed PLGA/β-TCP discs of five independent donors (N = 5). Individual data points shown are the mean of two technical replicates for each individual donor: donor D (●), donor E (▲), donor F (♦), donor G (■) and donor H (▼). Cells are cultured in basal medium (BM), osteogenic medium or osteogenic medium without β-glycerophosphate (BGP) for 28 days. Fold changes in **(A)** *ALPL,* **(B)** *ENPP1* **(C)** *ANKH* and **(D)** *PHOSHO1* expression at day 7, 14 and 28 are calculated according to the 2^−ΔΔCt^ method using *RPLP0* as the endogenous calibrator and the corresponding day 0 BM the normaliser. Two-way ANOVA is performed: *p < 0.5, * *p < 0.01.

The *ALPL*, *ENPP1*, *ANKH* and *PHOSPHO1* genes are related to phosphate cleavage and regulation during mineralisation of the extracellular matrix. The *ALPL* fold change expression cultured on 3D-printed discs under standard osteogenic condition shows a significant increase compared to BM from 0.26 to 1.34 at day 7 (*p* = 0.0313), from 0.14 to 1.98 at day 14 (*p* = 0.0195) and from 0.3 to 2.01 at day 28 (*p* = 0.0073) (Figure 6A). The subtraction of BGP leads to a slight trend of upregulation at day 7 and 28 and maintenance at day 14 compared to the osteogenic group. The fold change expression trend of *ENPP1* is downregulated in osteogenically driven cells for all time points compared to BM (Figure 6B). The subtraction of BGP recovers the downregulated trend of the osteogenic group to similar levels as the BM for all time points. The *ANKH* and *PHSOSPHO1* genes share the same expression profile pattern, which is also similar to the *ENPP1* gene expression profile. Osteogenically driven cells show a downregulated trend for all time points compared to BM (Figure 6C/D). The substruction of BGP recovers the downregulation of the osteogenic group to similar levels as the BM only at day 28 and remains downregulated at day 7 and 14.

The absence of exogenous BGP leads to an overall trend of upregulation of *ALPL, ENPP1, ANKH* and *PHOSPHO1* compared to the osteogenic group levels of hBM-MSCs cultured on 3D-printed PLGA/β-TCP discs and gene expression recovery close to BM levels in *ENPP1, ANKH* and *PHOSPHO1* gene expression.

### 3.6 Absence of exogenous BGP decreases free phosphate concentration in the conditioned media of hBM-MSCs cultured on 3D-printed PLGA/β-TCP discs

**FIGURE 7:**
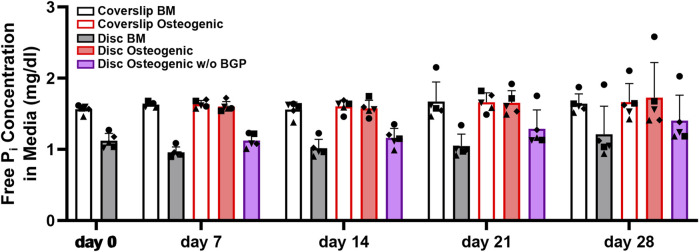
Free phosphate concentration in the media hBM-MSC cultured either on coverslips or 3D-printed PLGA/β-TCP discs of five independent donors (N = 5). Individual data points shown are the mean of two technical replicates for each individual donor: donor D (●), donor E (▲), donor F (♦), donor G (■) and donor H (▼). Cells are cultured in basal medium (BM), osteogenic medium or osteogenic medium without β-glycerophosphate (BGP) for 28 days. Conditioned media samples are collected at day 0, 7, 14, 21 and 28.

The measurement of free phosphate concentration in the conditioned media of hBM-MSCs of five independent donors cultured on 3D-printed discs reveals a strong increased trend in osteogenic medium with an average of 1.64 ± 0.15 mg/dL compared to BM with an average of 1.07 ± 0.17 or osteogenic medium without BGP (average of 1.24 ± 0.2 mg/dL) for all measured timepoints (Figure 7). The unconditioned BM is measured at 1.57 mg/dL, osteogenic medium at 1.68 md/dL and osteogenic medium without BGP at 1.54 mg/dL. The osteogenic group remains stable, while the BM group and osteogenic medium without BGP group are decreased compared to their corresponding unconditioned media.

The absence of exogenous BGP decreases the free phosphate levels in the conditioned media compared to the osteogenic group of hBM-MSCs cultured on 3D-printed PLGA/β-TCP discs and keeps them reduced over the period of the experiment.

### 3.7 Absence of exogenous BGP leads to similar stained area of mineral deposition of hBM-MSCs cultured on 3D-printed PLGA/β-TCP discs under osteogenic conditions

**FIGURE 8:**
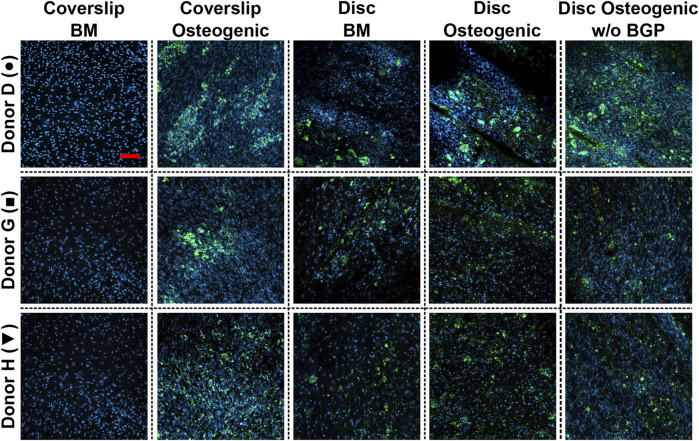
Images of bone cell mineralisation of hBM-MSC cultured either on coverslips or 3D-printed PLGA/β-TCP discs of three representative donors: donor D (●), donor G (■) and donor H (▼) stained OsteoImage™ (green) and DAPI (blue). Cells are cultured in basal medium (BM), osteogenic medium or osteogenic medium without β-glycerophosphate (BGP) for 28 days. Fluorescent confocal images are taken at day 28, scale bar: 200 µm.

Mineral deposition of hBM-MSCs of five independent donors cultured on 3D-printed discs for 28 days is stained using the OsteoImage™ Mineralisation Assay. The three representative donors (donor D, G and H) show visible stained area of mineralisation in all images under the three culture conditions when cells are cultured on 3D-printed discs (Figure 8). No differences in staining can be detected between these groups. The number of nuclei are visibly increased, which is in accordance with the increased DNA content of Figure 3C β-TCP from the 3D-printed disc and hydroxyapatite secreted by the differentiated cells are not distinguishable from each other.

The absence of exogenous BGB leads to a visibly similar stained area of mineral deposition and number of nuclei of hBM-MSCs cultured on 3D-printed PLGA/β-TCP discs compared to BM and osteogenic medium. Applying the osteogenic cocktail to cells cultured on coverslips leads to higher secretion of mineral deposition.

## 4 Discussion

The purpose of this study was to investigate whether β-TCP embedded within 3D-printed PLGA/β-TCP scaffolds can serve as an effective phosphate source during the osteogenic differentiation of hBM-MSCs and replace organic BGP as part of the osteogenic cocktail. The results of the cytotoxicity and thorough osteogenic *in vitro* experiments by examining cell activity, proliferation, and key osteogenic markers of hBM-MSCs cultured on 3D-printed discs show that BGP supplementation can have detrimental effects in a donor dependent manner. The results clearly indicate that cells can undergo osteogenesis with and without the supplementation of BGP when the scaffold contains β-TCP. Consequently, the use of exogenous BGP is redundant when cells are cultured on a phosphate-based material.

Despite the dose dependent decline in cell metabolic activity upon supplementation of BGP, the activity remains above 70% and therefore considered non-cytotoxic according to the ISO 10993-5 guidelines (Wallin and Arscott, 1998). BGP is known to be cytocompatible when cells are cultured on CCP. However, the data suggests that while viable, cells from some donors may be stressed in the presence of exogenous BGP when cultured on a phosphate-based material. The negative outcome of cells exposed to BGP when cultured on a phosphate-based material might be attributed to their exposure to high phosphate concentrations, which previously has been shown to have detrimental effects for BM-MSCs (Liu et al., 2009; Schäck et al., 2013). Previous studies have already demonstrated the advantages of replacing exogenous BGP with alternative phosphate sources such as inorganic Na_x_H_3-x_PO_4_, polyP nanoparticles and sodium phosphate (Schäck et al., 2013; Hatt et al., 2019; Rui et al., 2022). 10 mM BGP supplementation has been shown to cause non-physiological fluctuations of extracellular phosphate levels. Replacing BGP with inorganic Na_x_H_3-x_PO_4_ resulted in improved quality of mineralised matrix closer to hydroxyapatite, demonstrated by the ratio between calcium and phosphate (Schäck et al., 2013). The hypothesis that excessive quantities of phosphate results in the disruption of hBM-MSC homeostasis and consequent reduction in cellular activity appears to be donor dependent. Different donors exhibited varying responses in terms of ALP activity and proliferation under osteogenic conditions with and without BGP. These donors can be loosely classified into two groups: 1) those less affected and 2) those detrimentally affected by BGP supplementation. To demonstrate donor responses of the two groups, we divided the presented data into two Figures (Supplementary Figure 1/2) only containing the two osteogenic groups for comparison. ALP activity normalised to the DNA content of Donor D and F seems to be less disrupted by the supplementation with the traditional osteogenic cocktail (group 1), while donor E, G and H seem to be much more affected (group 2). Donor variability is also evident in the varying levels of ALP staining intensity observed among representative donors. Donors from group 1 were 48 and 51 years old at the time of the bone marrow donation and the donors from group 2 were 61, 74 and 71 years old. Donors from group 1 might have been in their premenopausal phase, which could arguably have an influence on the condition of the isolated MSCs (Zhu et al., 2009; Horinouchi et al., 2020), although further investigation with more donors is needed to draw a definitive conclusion. Replacing BGP with polyP nanoparticles has been shown to provide stable means for inducing osteogenic responses and reduce inter-donor variability in osteogenesis assays of hBM-MSCs (Hatt et al., 2019). Donor variability is commonly associated with the use of primary MSCs and can limit the statistical significance of findings, an important limitation in this study.

The removal of BGP has a positive effect on genes involved in matrix production such as *COL1A1*, *IBSP* and *SPP1* and supports the assumption that BGP supplementation might negatively influence the osteogenic differentiation of hBM-MSCs when cultured on a phosphate-based material. The early osteogenic marker, *COL1A1*, an important driver for collagen 1 production, is highly expressed in differentiated osteoblasts (Chen et al., 2021; Mollentze et al., 2021). BGP removal leads to a substantial upregulation and recovery of COL1A1, particularly in donors belonging to group 2. The average expression of late osteogenic markers *IBSP* (bone sialoprotein encoding gene) and *SPP1* (osteopontin encoding gene), which are involved in matrix production, do not appear to be strongly influenced by the removal of BGP. However, each donor exhibits largely different gene expression patterns for *IBSP* and *SPP1* under standard osteogenic conditions, which leads to a substantial inter-donor variance. The variance can be diminished when BGP is removed, partially evident in donors from group 2.

The expression of the transcription factor *SP7*, which plays a crucial role in the maturation of preosteoblasts and their differentiation into osteoblasts (Komori, 2006; Pokrovskaya et al., 2020), not only shows an increase in the group without BGP supplementation, but also displays a reduced variance similar to the gene expression profiles of *IBSP* and *SPP1*. *RUNX2*, a transcription factor essential for osteoblast differentiation (Komori, 2018), has been shown to display minimal differences in osteogenesis of hBM-MSCs (Loebel et al., 2015; Hatt et al., 2019). However, the downregulation of the chondrogenic marker *SOX9* makes it an indirect marker for osteogenesis, therefore the RUNX2/SOX9 ratio can be used as an osteogenic marker (Loebel et al., 2015). The subtraction of BGP does not influence the RUNX2/SOX9 ratio. The gene expression of *PPARγ* indicates off-target differentiation of hBM-MSCs into adipocyte-like cells under osteogenic conditions. This phenomenon is a common characteristic of hBM-MSCs and is induced by the presence of dexamethasone, which activates the glucocorticoid receptor (Della Bella et al., 2021). The continuous increase of *PPARγ* expression observed under standard osteogenic treatment could potentially be attributed by the presence of glycerol that is cleaved from the BGP.

To deepen our understanding of the impact of removing BGP from the osteogenic cocktail and highlight its redundancy, we investigated the gene expression of phosphate-related genes such as *ALPL*, *ENPP1*, *ANKH* and *PHOSPHO1,* genes involved in phosphate cleavage and regulation. *ALPL* is responsible for encoding the membrane-bound ALP. ALP is a commonly used osteogenic marker to show osteogenic differentiation of MSCs (Yeniyol and Ricci, 2018; Trivedi et al., 2020) and plays a crucial role in releasing phosphate by cleaving the phosphate ester bond (Levitt et al., 2022). Cells cultured on a phosphate-based material seem to show a delay in *ALPL* peak expression. Ectonucleotide pyrophosphatase/phosphodiesterase family member 1 (E-NPP1), encoded by the *ENPP1* gene, plays an important role in maintaining the balance of bone mineralisation (Gao et al., 2018). Its expression is vital for osteogenic differentiation of preosteoblastic cells (Nam et al., 2011). E-NPP1 is known to increase the levels of extracellular pyrophosphate (Nam et al., 2011), a well-established inhibitor for hydroxyapatite formation (Roberts et al., 2019). However, the use of exogenous pyrophosphate has been shown to stimulate the expression of osteogenic genes in osteoblastic MC3T3 cells (Pujari-Palmer et al., 2016). *ENPP1* gene expression has been reported to play a key role in the process of osteoinduction by CaP ceramics on hBM-MSCs (Othman et al., 2019). However, the fundamental role of E-NPP1 in MSCs is not extensively studied. Progressive ankylosis protein homolog (ANK), encoded by the *ANKH* gene, is highly expressed in osteoblasts (Szeri et al., 2020). It is involved in the osteogenic fate decision of adult mesenchymal precursor cells and its absence has been shown to favor adipogenesis (Minashima et al., 2017). *ANKH* is used as a osteogenic marker of BM-MSCs due to its association with ALP (Granchi et al., 2010). Phosphoethanolamine/phosphocholine phosphatase, encoded by the PHOSPHO1 gene, is a matrix vesicle phosphatase that is involved in skeletal mineralisation (Roberts et al., 2007; Dillon et al., 2019). It generates inorganic phosphate (Hsu et al., 2022), essential for the mineralisation process. The downregulation of *ENPP1, ANKH* and *PHOSPHO1* gene expression observed when cells are cultured on a phosphate-based material upon osteogenic treatment in contrary to the coverslip suggest an active role of the material itself on the expression of these genes. However, the mechanism by which β-TCP from 3D-printed scaffolds can be cleaved remains to be investigated. These results support the theory that hBM-MSCs are sensitive to high concentrations of phosphate and suggest that the supplementation of BGP may overwhelm the cells when they are already exposed to a phosphate-based material. The delicate sensitivity of osteoblastic cells to exogenous phosphate has been shown in previous studies, in which high levels of inorganic phosphate induced cell apoptosis (Meleti et al., 2000; Liu et al., 2009). Our findings suggest that the regulation of matrix and phosphate-related genes are influenced by the presence of BGP, and together with the cell metabolic activity measurements, caution should be exercised when using BGP supplementation.

Interestingly, the decrease in gene expression profile of phosphate-related genes upon osteogenic treatment shows the opposite trend compared to the increased concentration of free phosphate in the media. The inactivity of these genes under osteogenic treatment indicates that the cells control the excessive amounts free phosphates in the media by their downregulation. hBM-MSCs undergoing osteogenic differentiation cleave the BGP nearby their membrane, releasing free phosphate to the surrounding medium, thereby maintaining a high concentration of free phosphate. In contrast, cells cultured without the supplementation of BGP have an increased expression of these phosphate-related genes but result in the decline of free phosphate in the medium. This phenomenon suggests that the cells that are cultured on the CaP material without BGP supplementation uptake the cleaved free phosphate from the 3D-printed PLGA/β-TCP disc locally, without secreting free phosphate into the surrounding medium. Consequently, high concentration of free phosphate in the medium is prevented. In summary, ALP activity and proliferation results, along with the gene expression profile of matrix-related,—transcriptional—and phosphate-related genes, supports the assumption that BGP supplementation has a detrimental impact on osteogenic differentiation of hBM-MSCs when cultured on a phosphate-based material.

Additional limitations encountered in this study is the difficulty of measuring mineralised deposition of the cells. Common staining protocols such as Alizarin Red or Von Kossa, which are used to visualize and quantify cell-secreted hydroxyapatite, are strongly positive to any CaP containing materials. This circumstance increases the challenge in distinguishing mineralisation of the underlying material from the matrix secreted by the cells. Therefore, the application of these histological methods cannot be used for such experiments. Chemical analysis of the material at the element level using techniques like energy-dispersive X-ray spectroscopy (EDX) or Fourier transform infrared spectroscopy can indirectly confirm the presence of hydroxyapatite by measuring the CaP ratio (Langenbach and Handschel, 2013; Schäck et al., 2013). However, these methods present their own technical difficulties. Uneven densities in the PLGA/β-TCP blend does not allow for accurate measurement using the EDX method. Quantification calcium concentration has been employed as a method to indicate mineralisation (Kuss et al., 2018; Ebrahimi et al., 2022). However, the chemical interaction between free phosphate and the material and consequential precipitation of CaP can give false positive results. *In vitro* fluorescence imaging using the OsteoImage™ Mineralization Assay is specifically designed to stain the hydroxyapatite portion of bone-like nodules deposited by cells. Unexpectedly, we discovered positive staining of the OsteoImage™ dye on cell-free 3D-printed β-TCP discs (images not shown). This occurrence weakens the interpretation of the presented confocal images. The investigation of mineral output has always been a crucial marker for osteogenesis. However, due to the nature of the CaP-based material, accurately quantifying cell-secreted mineral deposition remains a limitation in this study. Necessary controls must be included, and caution must be taken when testing biomaterials as artifacts can alter data interpretations. Further research and alternative methods may be necessary to overcome this limitation and provide a more comprehensive analysis of mineralisation in this context.

The use of the osteogenic cocktail has been specifically defined for 2D cell culture systems and cannot be easily translated into complex 3D matrices systems. The findings presented in this study emphasise the importance of investigating the optimal osteogenic culture medium to differentiate MSCs when combined with phosphate-based materials for bone tissue engineering. The removal of BGP from the medium enhances the resemblance of a cell-laden bone substitute to the *in vivo* situation, thereby improving the validation of biomaterials for their osteogenic potential. The osteogenic cocktail is a potent osteogenic inducer that pushes MSCs to differentiate into osteoblasts even in an unnatural environment such as CCP or coverslips, that would not be expected to osseointegrate *in vivo*. In this experimental *in vitro* set-up, bioinert plastics exhibit osteoconductive properties. Therefore, conducting an osteogenic differentiation experiment using MSCs cultured on plastic or coverslips should primarily serve as negative a control. An important aspect in bone tissue engineering is to assess whether a biomaterial is suitable to be used as a bone substitute *in vivo* and investigate the effects on neighboring tissues and cells. The positive effects of β-TCP on adjacent cells have reported in literature. Higher content of β-TCP in ceramic scaffolds lead to increased secretion of vascular endothelial growth factor from hBM-MSCs, which enhanced endothelial cell proliferation and capillary formation in a co-culture *in vitro* experiment (Pereira et al., 2019). β-TCP-granules lead to early vascularization and bone healing when endothelial progenitor cells and hBM-MSCs were delivered upon *in vivo* implantation (Seebach et al., 2010). Furthermore, the *in vivo* implantation of pre-vascularized tissue engineered bone grafts based on β-TCP resulted in the acceleration of vascular formation (Xu et al., 2022) and vessel penetration into the scaffold (Xu et al., 2022). *In vitro* experiments have shown that osteoclasts can resorb β-TCP (Davison et al., 2014; Matsunaga et al., 2015), which would mimic the natural bone remodeling process *in vivo*. However, the *in vivo* interaction between hBM-MScs and osteoclasts or myoblasts in the presence of β-TCP is not well understood.

By minimizing the enormous difference between *in vitro* and *in vivo* studies, the translational potential of tissue engineered bone substitutes can be enhanced. Optimizing the osteogenic culture medium to create a scenario closer to the natural environment can contribute to reducing this disparity. We therefore propose that researchers consider removing BGP from their osteogenic medium when testing phosphate-based bone substitutes using MSCs. By addressing these considerations and refining the experimental conditions to better mimic the *in vivo* environment, researchers can improve the relevance and applicability of their findings in the field of bone tissue engineering.

## 5 Conclusion

The presented results provide evidence for osteogenic differentiation of hBM-MSCs cultured on 3D-printed PLGA/β-TCP discs with and without the supplementation of BGP in the osteogenic medium. Our findings suggest that hBM-MSCs can use the β-TCP embedded within a 3D-printed scaffold as a phosphate source, eliminating the need for exogenous BGP. Nevertheless, the mechanisms by which the phosphate is cleaved remains to be investigated. Furthermore, we demonstrated the donor dependent negative impact of BGP on various aspects including cell metabolic activity, ALP activity, proliferation, and gene expression of osteo- and phosphate-relevant markers when cells are cultured on a phosphate-based material. Understanding the interaction between cells and 3D-printed scaffolds can help tailor the design and composition of the scaffold. Thus, leading to a pro-osteogenic environment that supports cell differentiation and infiltration with the aim of improving successful translation of such bone substitutes to *in vivo* bone defect repair.

## Figures and Tables

**Table:** 

**ALP**	Alkaline phosphatase
**BGP**	β-glycerophosphate
**BM**	Basal medium
**β-TCP**	β-tricalcium phosphate
**CaP**	Calcium phosphate
**CCP**	Cell culture plastic
**CTB**	CellTiter-Blue^®^
**EDX**	Energy-dispersive X-ray spectroscopy
**FBS**	Foetal bovine serum
**HAp**	Hydroxyapatite
**hBM-MSCs**	Human bone marrow-derived mesenchymal stromal cells
**PBS**	Phosphate buffered saline
**PCL**	Polycaprolactone
**PEN/STREP**	Penicillin/Streptomycin
**PLGA**	poly (lactic-co-glycolic acid)

## Data Availability

The raw data supporting the conclusion of this article will be made available by the authors, without undue reservation.
