# Supplementary figures and images for: β-TCP from 3D-printed composite scaffolds acts as an effective phosphate source during osteogenic differentiation of human mesenchymal stromal cells
Source: Front Cell Dev Biol. 2023 Oct 26;11:1258161. doi: 10.3389/fcell.2023.1258161 (PMC10641282; doi:10.3389/fcell.2023.1258161)

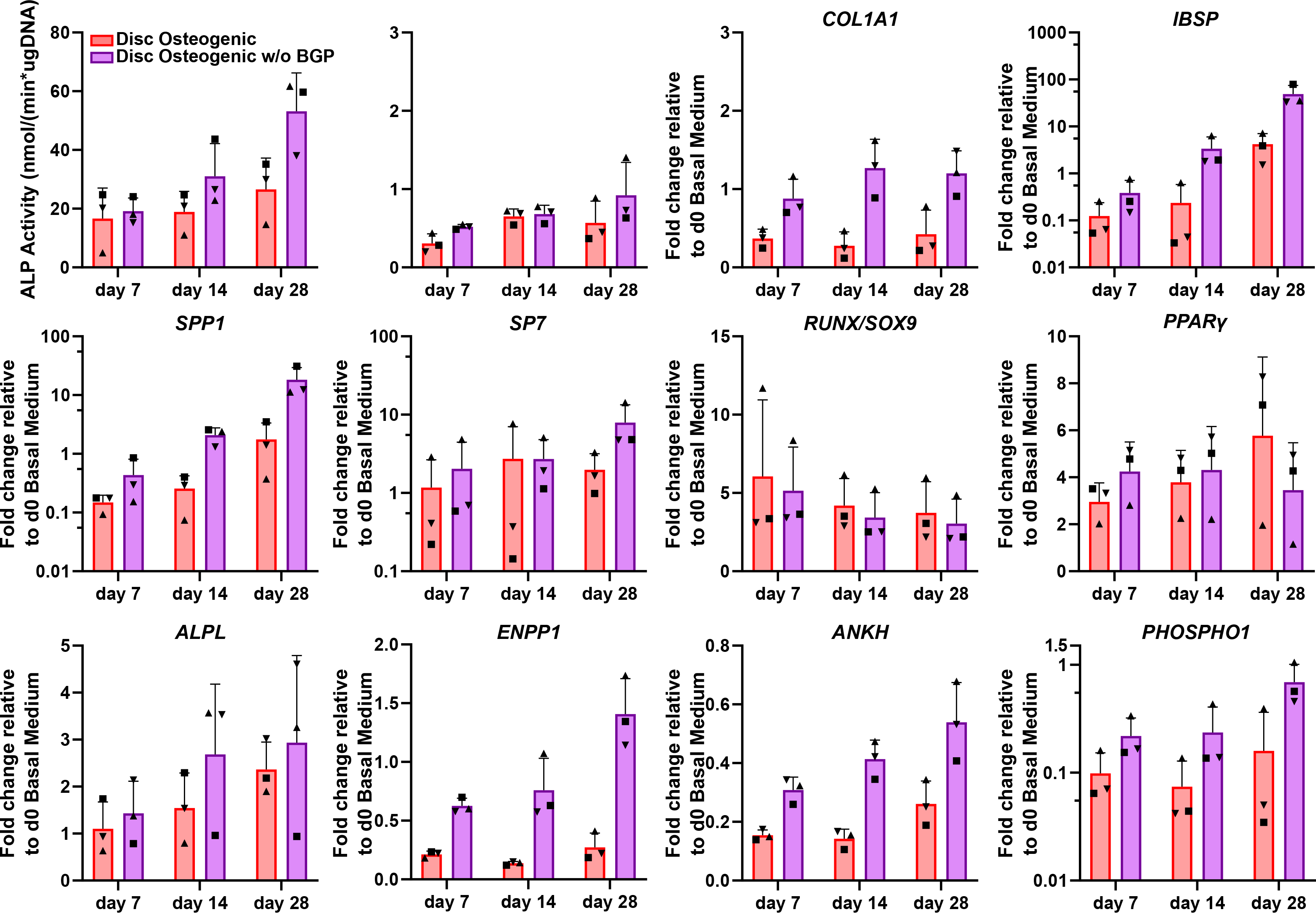

Supplement: Supplementary file 1 [file Image2.TIF]

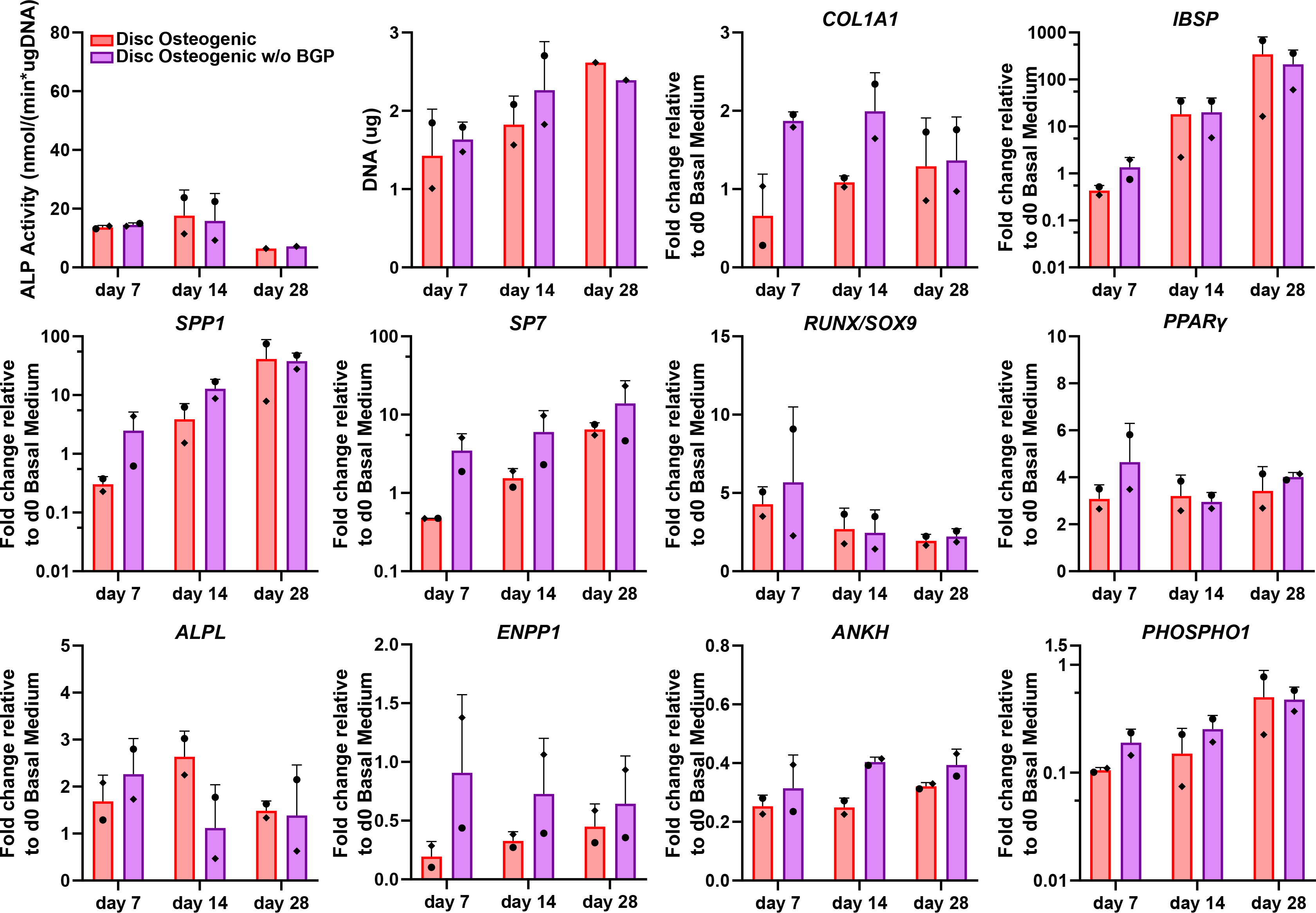

Supplement: Supplementary file 2 [file Image1.TIF]
